# Allergy to the fistula needle: a rare cause of allergy in hemodialysis (a case report)

**DOI:** 10.11604/pamj.2024.49.69.43847

**Published:** 2024-11-08

**Authors:** Meriam Khadhar, Rania Kendil, Sarra Hadded, Hanene Gaied, Raja Aoudia, Asma Bettaieb, Mouna Jerbi, Rym Goucha

**Affiliations:** 1 Department of Nephrology, Dialysis and Kidney Transplantation, Mongi Slim Hospital, Tunis, Tunisia,; 2 Faculty of Medicine, University of Tunis El Manar, Tunis, Tunisia,; 3 Laboratory of Kidney Disease (LR00SP01), Tunis, Tunisia

**Keywords:** Hemodialysis, anaphylaxis, di-(2-ethylhexyl) phthalate, arteriovenous fistula, case report

## Abstract

Anaphylactic reactions are rare but potentially life-threatening events that may occur during medical procedures. This present case highlights a distinct occurrence of an anaphylactic reaction in a patient undergoing hemodialysis, specifically induced by the utilization of a fistula needle along with a review of the literature.

## Introduction

The prognosis of renal failure has undergone a revolutionary transformation since 1942 with the discovery of an extracorporeal blood epuration technique commonly referred to as hemodialysis [1]. It is the most common form of kidney replacement technique which has increased survival rates and improved the quality of life for over one million people [2]. However, the extensive array of materials, both solid and liquid in contact directly with human blood supports the occurrence of clinical and subclinical allergic reactions, serving as evidence of biocompatibility [3,4].

## Patient and observation

**Patient information:** we present a case involving a 38-year-old female with a medical history of type 1 diabetes, arterial hypertension, and asthma. She has chronic kidney disease at the stage of hemodialysis, related to diabetic nephropathy. She had received thrice-weekly maintenance hemodialysis for four months using a femoral catheter before transitioning to an arterio-venous fistula. The dialysis has proceeded without any issues. Her dialysis routine consisted of the use of AK 48 Dialyser, blood tubing set (steam sterilized, di-2-ethyl hexylphthalate free), fistula needle (steam sterilized, containing di-2-ethyl hexylphthalate), and polyflux filter (steam sterilized).

**Clinical findings:** thirty minutes into the dialysis procedure following insertion of the fistula needle, the patient experienced a sudden onset of symptoms including, facial flushing, dyspnea, and hypotension. The patient was promptly administered 200 mg of hydrocortisone hemisuccinate intravenously and 100 ml of saline solution. After the administration of emergency medications, the patient's symptoms gradually diminished, and vital signs stabilized.

**Diagnostic assessment:** further investigations revealed no evidence of infection or other systemic triggers for the anaphylactic reaction. The complete blood count indicated eosinophilia at a measurement of 3000/mm^3^. A review of her previous laboratory results showed no hypereosinophilia (eosinophilic count below 500/mm^3^). No new medication has been introduced in the past few weeks. Following this clinical overview, the diagnosis of anaphylactic reaction was retained.

**Therapeutic intervention:** during the subsequent session, we transitioned to a different dialysis machine. We also changed the dialyzer. The blood tubing set was flushed with two liters of saline solution (twice the usual saline). Regardless, the patient displayed the same anaphylactic reaction as observed in the previous session. Her symptoms resolved with stopping dialysis and 200 mg of Hydrocortisone hemisuccinate. Due to our suspicion of an anaphylactic reaction, we refrained from conducting blood restitution. Given the temporal relationship between needle insertion and the onset of symptoms, we suspected an allergic reaction to the fistula needle. For the next two sessions, her access was switched back to a femoral catheter, and these sessions were conducted without any incidents. As a result, the subsequent session was conducted by flushing the fistula needle with 150 milliliters of saline solution and rinsing the blood tubing set by two liters of saline solution.

**Follow-up and outcome:** the patient was closely monitored for any signs of systemic involvement, and during the whole session she did not experience any additional adverse reactions, and her hemodialysis treatments were continued without complications. For the next two months, we used the same protocol of preparation of the dialysis circuit including 2 liters priming of the blood tubing set and 150 ml fistula needle rinsing. The patient did not experience any complications, and the complete blood count revealed normal levels of eosinophils (280/mm^3^).

### Patient perspective

**Day 1:** “today's dialysis session started like any other, with me feeling a mix of apprehension and routine. As a 38-year-old woman with type 1 diabetes, high blood pressure, and asthma, dialysis has become a regular part of my life. I've grown accustomed to the hum of the machines and the steady rhythm of the procedure. But thirty minutes into today's session, everything changed. Suddenly, I felt a wave of heat wash over me, my face flushing, and my breathing becoming labored. Panic set in as I struggled to catch my breath, and my blood pressure dropped alarmingly. It was terrifying, to say the least. The medical team acted swiftly, administering medication and fluids to stabilize me. Slowly, the symptoms eased, but the fear lingered. What had caused this sudden reaction? I couldn't shake the feeling of vulnerability, wondering if it could happen again”.

**Day 2:** “tests revealed no clear culprit for my ordeal. No infections, no new medications-just unanswered questions. The diagnosis of anaphylaxis hung over me like a dark cloud, its unpredictability casting a shadow on future treatments”.

**Day 3:** “as we prepared for another session, there was a palpable tension in the air. Would history repeat itself? Despite precautions and changes to the equipment, the dreaded reaction reared its head once more. This time, the suspicion fell on the very tool meant to help me-the fistula needle. It was a cruel irony, realizing that the source of my treatment could also be the source of harm”.

**Day 4:** “with each subsequent session, fear gnawed at me. Would today be the day it all went wrong again? But gradually, as the weeks passed without incident, a cautious optimism began to take root. The adjustments to the procedure seemed to be working, and with each uneventful session, my confidence grew”.

**Months later:** “looking back now, it's hard to believe how far we've come. The initial terror has faded, replaced by a sense of resilience. Dialysis is still a part of my life, but it no longer looms over me like a specter of fear. Thanks to the diligence of my medical team and the adjustments made to my treatment plan, I can finally breathe a little easier”.

**Informed consent:** the patient provided their informed consent for publication of this case.

## Discussion

Hemodialysis enhances patients´ quality of life and reduces morbidity and mortality. However, a significant challenge arises from the occurrence of intra-dialytic complications, with particular emphasis on hypersensitivity reactions [3]. Four types of hypersensitivity reactions have been identified by the Gell and Coombs classification [5]. Type I or anaphylactic response is an immediate IgE-mediated response. Depending on the type of allergen and the route of exposure, clinical presentation may vary from mild symptoms (rhinitis, conjunctivitis, and urticaria) to more severe symptoms (angioedema, asthma, anaphylactic shock). These reactions can occur immediately or within a few minutes to a few hours from allergen exposure. Type II is a cytotoxic-mediated response that can cause immune thrombocytopenia, autoimmune hemolytic anemia, and autoimmune neutropenia. The timing of the reaction is variable. Type III is mediated by immune complexes that deposit into tissues leading to serious organ damage such as vasculitis, glomerulonephritis, and arthritis. Type IV is a delayed cell-mediated reaction and can be life-threatening in some cases, such as DRESS or bullous exanthems.

Type I or IgE-mediated response is the most common reaction in hemodialysis. Several agents have been implicated in these reactions, including medications (such as heparin, erythropoiesis-stimulating agents, and iron), dialysis membranes, plasticizers such as di-2-ethyl hexyl-phthalate (DEHP), isocyanates derived from polyurethane potting materials, and sterilizing agents based on ethylene oxide (EO) [4,6]. In this case, all the materials in the dialysis extra-corporal circuit were steam sterilized which rules out the hypothesis of allergy to EO. The possibility of a drug allergy was considered, but the patient hasn´t been put under any new medication in the past three months, significantly reducing the likelihood of this hypothesis. We suspected an allergy to the dialyzer, but the patient exhibited the same reaction with another type of membrane. We dialyzed the patient on another dialysis machine, but she developed the same symptoms. Considering the chronological connection between the first use of the fistula needle and the allergic reaction with a delay of four months between the start of dialysis and the symptoms, an allergy to the fistula needle was suspected. The technical documentation for the fistula needle specified that the material underwent steam sterilization, and that di-2-ethyl hexylphthalate (DEHP) was employed as a plasticizer. However, it's important to note that the blood tubing set used was DEHP-free. According to a study conducted by Faouzi *et al*. in 1999, an approximate amount of 75.2 mg of DEHP was leached from the dialyzer during a solitary dialysis session with a range from 44.3 to 197.1 mg [7].

In our case, we suspected DEHP to be the causative agent given the proven link between phthalate exposure and allergic diseases [8]. Indeed, DEHP was the only component newly introduced right before the allergic reaction. Consequently, the fistula needle has been rinsed with 150 ml of saline solution before each dialysis session as there is no other alternative fistula needle available in our center.

Therefore, the patient did not experience any symptoms indicating an allergic reaction. This further supported our hypothesis that DEHP contained in the fistula needle was responsible for the allergic reaction. We searched for similar cases by reviewing previous studies to support our hypothesis. In the study conducted by Röckel *et al*. in 1989, DEHP was considered to play a minor role in dialysis-related hypersensitivity reactions [6]. However, the population included didn´t experience any severe allergic reaction at variance with our case. Furthermore, most case reports discussed the role of EO in allergic reactions during dialysis [6,9].

To explore the potential for allergic reactions during dialysis stemming from additional antigens, Dolovich *et al*. published a report in 1987 studying antibody responses to phthalates and formaldehyde [10]. A test for phthalates IgE antibodies using phthalic anhydride (PA) and human serum albumin (HAS) was performed on 71 chronic hemodialysis patients and 80 controls to ascertain if there is a potential for sensitization to it. The final results indicated the presence of elevated IgE antibody levels against PA-HAS in the blood serum of certain chronic hemodialysis patients. Further experiments demonstrated the specificity of these antibodies to PA-HSA, as they were able to inhibit the binding of PA-HSA IgE antibodies but not other types of antibodies tested. These findings highlight the importance of understanding and monitoring sensitization to phthalates in this population to mitigate potential allergic complications.

## Conclusion

While allergies to the fistula needle during hemodialysis are relatively rare, they pose a significant concern for patients with end-stage renal disease. Further research is needed to better understand the causes and mechanisms behind these allergies, allowing for the development of safer and more allergen-free materials for fistula needles.

## References

[1] Lohani S, Knicely DH, Knicely DH, Abdel-Rahman EM, Greenberg KI (2021). Chapter 1: Brief History of Home Hemodialysis. Handbook of Home Hemodialysis.

[2] Bello AK, Okpechi IG, Osman MA, Cho Y, Htay H, Jha V (2022). Epidemiology of haemodialysis outcomes. Nat Rev Nephrol.

[3] Davenport A (2006). Intradialytic complications during hemodialysis. Hemodial Int.

[4] Butani L, Calogiuri G (2017). Hypersensitivity reactions in patients receiving hemodialysis. Ann Allergy Asthma Immunol.

[5] Justiz Vaillant AA, Vashisht R, Zito PM (2024). Immediate Hypersensitivity Reactions. StatPearls.

[6] Röckel A, Klinke B, Hertel J, Baur X, Thiel C, Abdelhamid S (1989). Allergy to dialysis materials. Nephrol Dial Transplant.

[7] Faouzi MA, Dine T, Gressier B, Kambia K, Luyckx M, Pagniez D (1999). Exposure of hemodialysis patients to di-2-ethylhexyl phthalate. Int J Pharm.

[8] Bølling AK, Sripada K, Becher R, Bekö G (2020). Phthalate exposure and allergic diseases: Review of epidemiological and experimental evidence. Environ Int.

[9] Crane C, Cunard RA, Sweiss N, Scanlon N, Doherty TA, Potok OA (2023). Anaphylaxis From Ethylene Oxide-Sterilized Dialysis Tubing and Needles: A Case Report. Am J Kidney Dis.

[10] Dolovich J, Evans S, Baurmeister U, Schulze H, Ali M, Shimizu A (1987). Antibody responses to hemodialysis-related antigens in chronic hemodialysis patients. Artif Organs.

